# Cysteic acid grafted to magnetic graphene oxide as a promising recoverable solid acid catalyst for the synthesis of diverse 4*H*-chromene

**DOI:** 10.1038/s41598-020-77872-8

**Published:** 2020-12-01

**Authors:** Firouz Matloubi Moghaddam, Mohammad Eslami, Golfamsadat Hoda

**Affiliations:** Laboratory of Organic Synthesis and Natural Products, Department of Chemistry, Sharif University of Technology, Azadi Street, P.O. Box 111559516, Tehran, Iran

**Keywords:** Biochemistry, Chemistry

## Abstract

4*H*-chromenes play a significant role in natural and pharmacological products. Despite continuous advances in the synthesis methodology of these compounds, there is still a lack of a green and efficient method. In this study, we have designed cysteic acid chemically attached to magnetic graphene oxide (MNPs·GO-CysA) as an efficient and reusable solid acid catalyst to synthesize 4*H*-chromene skeletons via a one-pot three components reaction of an enolizable compound, malononitrile, an aldehyde or isatin, and a mixture of water–ethanol as a green solvent. This new heterogeneous catalyst provides desired products with a good to excellent yield, short time, and mild condition. This procedure presents an environmentally friendly approach for the synthesis of a great number of 4*H*-chromene derivatives.

## Introduction

**Figure 1:**
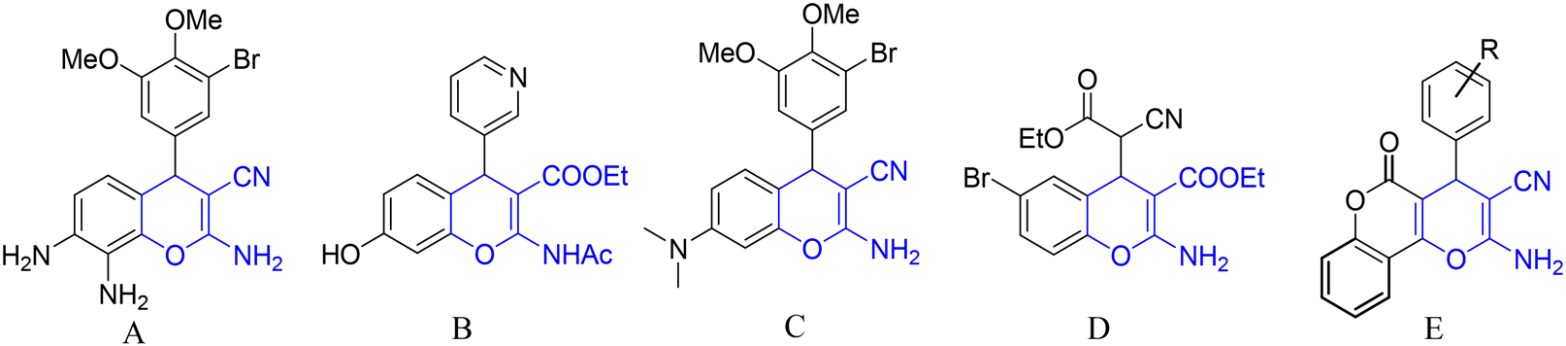
Structures of some 4*H*-chromenes possessing diverse biological activities.

4*H*-chromenes represent an important class of oxygen-containing heterocycles and a key building block of many natural products. They are also widely found in nature, in some edible fruits and vegetables, to name but a few, and these compounds also are broadly used as cosmetics and pigments^1–3^. 4*H*-chromene scaffolds exhibit pharmacological properties and biological activities such as antioxidant^4^, antimicrobial^5,6^, antiviral, antibacterial, pro-apoptotic^7–11^, anticancer^12^, antifungal, anticoagulant, antinociceptive^13^, antiproliferative^14^, antitubercular, antiallergic, antibiotic, hypolipidemic, and immunomodulating activities. They also are used as cognitive enhancers, in order to treat neurodegenerative disease^15,16^ such as Alzheimer’s disease, Huntington’s disease, amyotrophic lateral sclerosis, AIDS, Parkinson’s disease, Down’s syndrome, and also myoclonus and schizophrenia^17^. For example, 4*H*-chromene derivatives such as A, B, C, D, and E are known as apoptosis inducer, insulin-regulated aminopeptidase for enhancing memory, learning functions inhibitor, and anticancer therapeutic agents respectively, which are shown in Fig. 1^11,18–20^.

In this regard, multi-component reactions (MCRs) have gained considerable attention for constructing a broad range of complex molecules in a highly efficient, rapid, step-economic, low-cost, and eco-friendly manner^21–27^. Because of the benefits of MCRs and the great importance of 4*H*-chromenes, syntheses of these compounds have been developed using different catalysts in the multicomponent reaction between malononitrile (or ethyl cyanoacetate), a diverse electron-rich phenol or enolizable carbonyl compound, and an aldehyde^1^. Recent catalytic systems for the synthesis of 4*H*-chromene derivatives consist of Fe(HSO_4_)_3_^28^, nickel nanoparticles^29^, ZrO_2_ nanoparticles^30^, Zn_4_O(H_2_N-TA)_3_^31^, ZnS nanoparticles^32^, nano-sized MgO^33^, CoFe_2_O_4_^34^, CuO-CeO_2_^35^, egg shell^36^, chitosan^37^, polymer-supported palladacycles^38^, [2-aemim][PF_6_]^39^ and TMG-[bmim][X]^40^ under microwave radiation, [bmim]OH^41^, IL-HSO_4_@SBA-15^42^, hexadecyltrimethylammonium bromide^43^, l-proline^44^, l-proline-melamine^45^, tetraalkylammonium halides^46–49^, SB-DBU.Cl^50^, potassiumphthalimide-*N*-oxyl^51^, potassium phthalimide^52^, sodium selenate^53^, sodium alginate^54^, Sodium ethylene diamine tetraacetate^55^, morpholine^56^, 4-dimethylamino- pyridine^57^, 4-DMAP^58^, piperidine^18,59^, piperidinium acetate^60^, (DHQD)_2_PYR^61^, tungstic acid functionalized mesoporous SBA-15^62^, 1,8-diazabicyclo[5.4.0]undec-7-ene^63^, glycine^64^, imidazole^65^, heteropolyacid^66^, meglumine^67^, Mg/Al hydrotalcite^68^, PEI@Si–MNP^69^, PEG-SO_3_H^70^, alumina^71^, nano-sized zeolite clinoptilolite^72^, Nickel Nanoparticles^29^, (CTA)_3_[SiW_12_]-Li^+^-MMT^73^, PMO-ICS^74^, poly(*N*,*N*′-dibromo-Nethyl-benzene-1,3-disulfonamide (PBBS)^75^, KSF^76^, combined NaOAc/KF^77^, MeSO_3_H^78^, TiCl_4_^79,80^, protic ionic liquid^81^, Bmim(OH)/20 mol% chitosan^82^, MA liquid-phase^83^, Bovine Serum Albumin^84^, and KF alumina^85^.

However, lots of the mentioned catalysts suffer from disadvantages such as environmental pollution, high cost, the difficulty of catalyst removal, and demanding harsh reaction conditions. According to the importance and the broad application of these types of heterocyclic compounds, there is still a great demand for a more feasible, easy, green, and efficient way to synthesize these compounds.

Catalysis is a key to the green chemistry gates. In this regard, the designation of a benign, reusable, and efficient catalyst can provide us with a green approach. Magnetic nanoparticles (MNPs), due to their good dispersion and ease of separation, can be considered as great applicable catalysts in both lab and industrial scales^86,87^. In this study, we designed a magnetic nano-scaled catalyst which has been functionalized by cysteic acid to improve the catalytic activity. By introducing such a catalyst, we can take advantage of both functional groups of graphene oxide and sulfone groups, the broad surface of graphene structure, and ease of separation of a magnetic catalyst.

One of the technical difficulties of typical catalysts is the separation of catalyst off the reaction batch after product gaining. Separation techniques such as filtration and centrifugation are used in such catalysts while magnetic nanoparticles are clearly much more convenient to separate because of their response to an external magnetic field. Using MNPs as catalyst provides the reaction with an effective and rapid way of catalyst separation, making the technique efficiently applicable to both industrial and lab-scale syntheses^88–90^.

**Figure 2:**
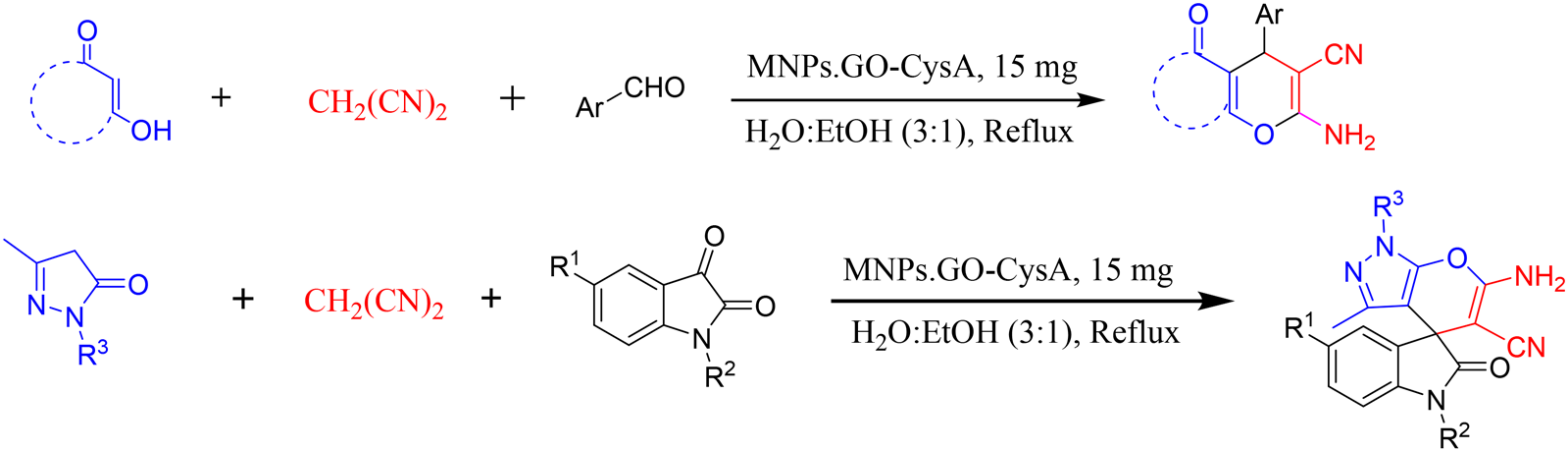
One-pot three-component reaction of enolizable compound, active methylene nitriles, and aldehydes catalyzed by MNPs·GO-CysA in water:ethanole.

To sum up, Graphene Oxide (GO) is the product of chemical exfoliation of graphite, which is an oxygenated monolayer graphene platelet. It contains plentiful of functional groups including hydroxyl, carboxyl, epoxy, and carbonyl group^91^. As a result of having mentioned functional groups, graphene Oxide with its open π-electron system can be easily functionalized by appropriate organic or inorganic molecules. Additionally, GO’s 2D structure provides the catalyst system with a high surface area and an excellent mechanical strength^92–96^. Due to the increasing demand for environmentally friendly synthetic processes, using heterogeneous catalysts is getting importance^97–104^. l-cysteic acid, which is an amino acid with a C-terminal sulfonic acid group, can be effectively used as a solid acid catalyst. By magnetizing Graphene Oxide and functionalizing it via an environmentally friendly, bio-degradable Lewis acid, we herein present a new heterogeneous, efficient, easily separable, with a high effective surface available catalyst for synthesizing 4*H*-chromene derivatives (Fig. 2). In general, we took advantage of cysteic acid and graphene oxide as active sites of the catalyst and immobilized these sites on nanomagnetic Fe_3_O_4_, which are bind together via a covalent bond^105,106^.

## Results and discussion

**Figure 3:**
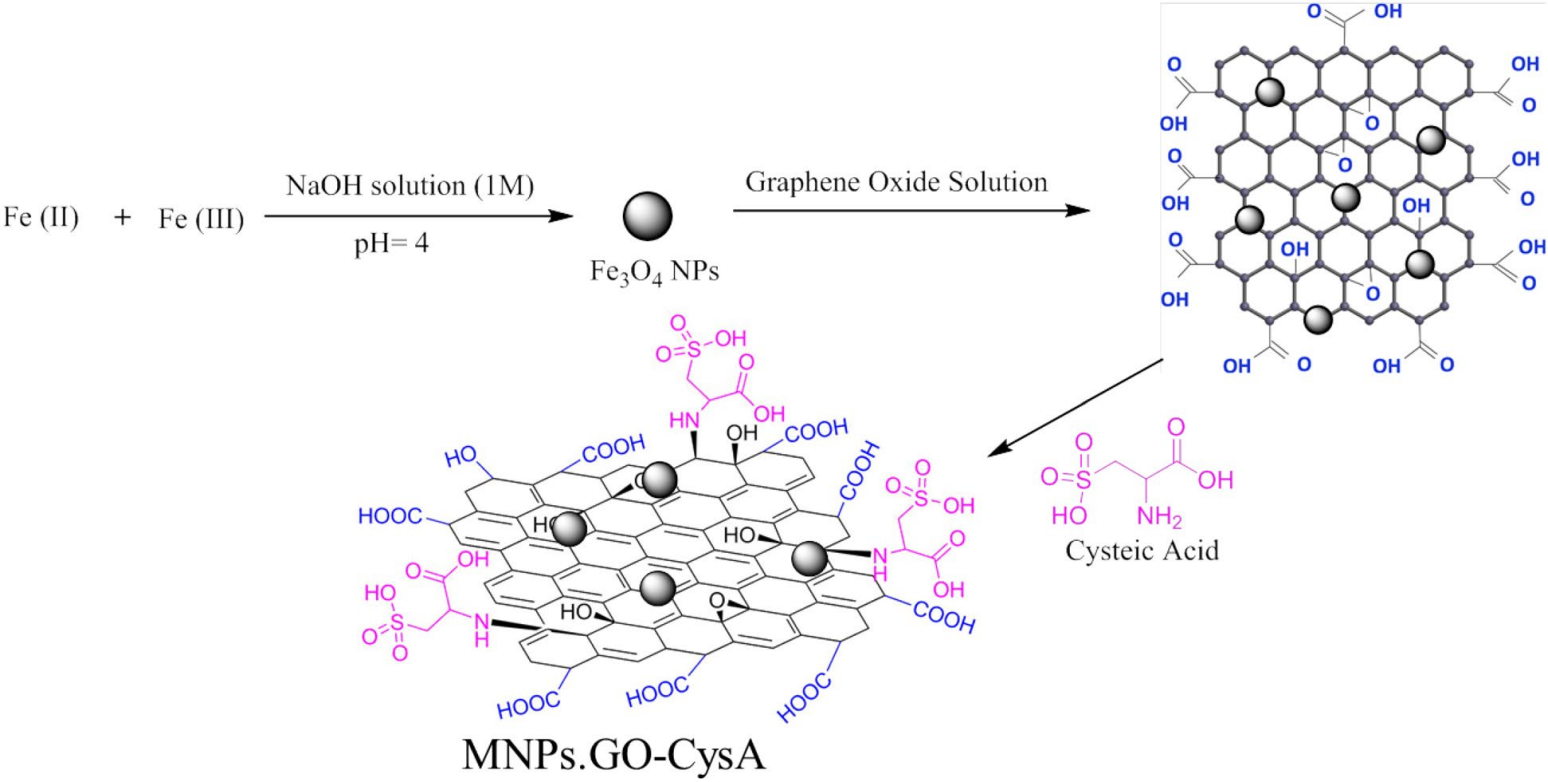
The schematic pathway for synthesis of MNPs·GO-CysA.

The MNPs·GO-CysA catalyst was synthesized using a few steps presented in Fig. 3. Details of the preparation method are described in the experimental section.

**Figure 4:**
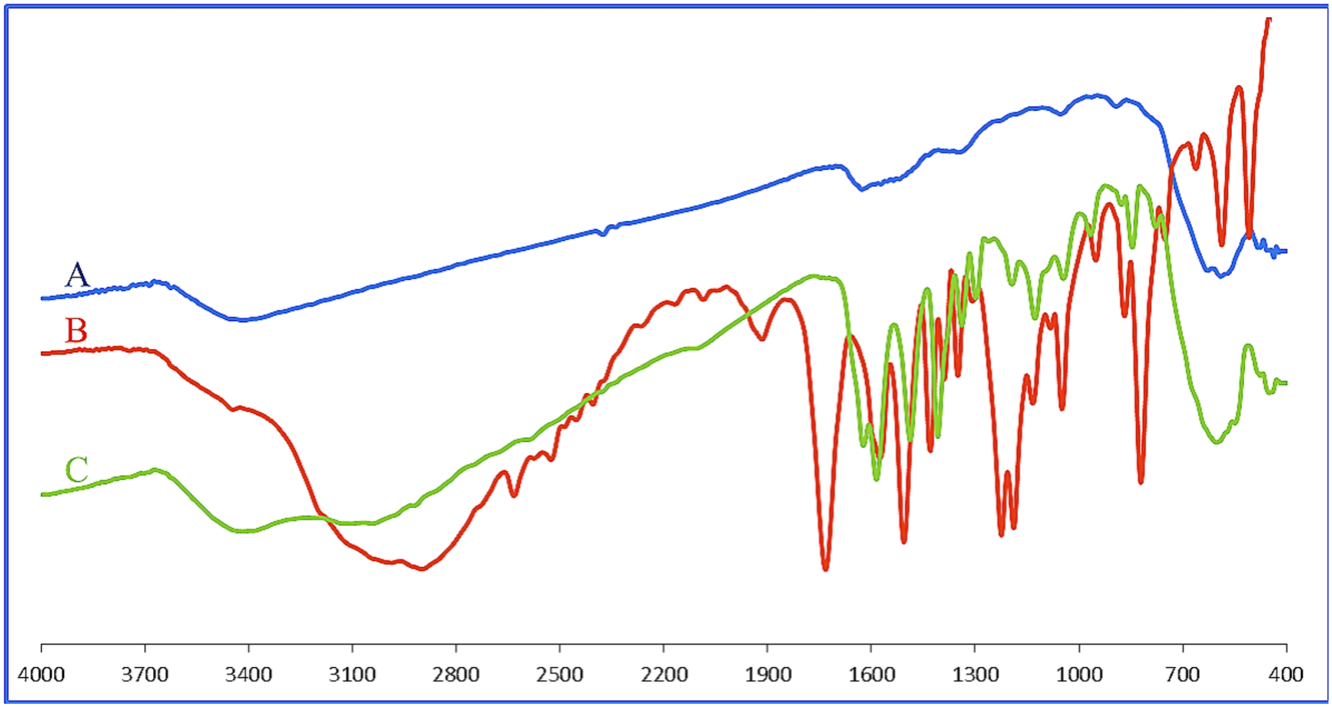
The FTIR spectrums of (**A**) Fe_3_O_4_, (**B**) Cysteic acid, and (**C**) MNPs·GO-CysA.

The FTIR spectrum is shown in Fig. 4. demonstrates the formation of desired bonds and the presence of new functional groups of MNPs·GO-CysA. The band at 640 cm^−1^ is attributed to the Fe–O bond vibration as proof of the existence of Fe_3_O_4_ in MNPs·GO-CysA^107^. The intense broad bands at ~3400 can be attributed to stretching of O–H in GO, Fe_3_O_4_ , and the sulfonic group of cysteic acid^108^. The peaks that appeared at 1137 cm^−1^ and 1026 cm^−1^ are due to the SO_2_ asymmetrical and symmetrical stretching vibrations^109^. In addition to the previous note, the absorption peak at 854 cm^−1^ correspondings to bonded N–H stretching, confirmed the formation of a chemical bond between cysteic acid and the magnetic GO sheets (Fig. 4).

**Figure 5:**
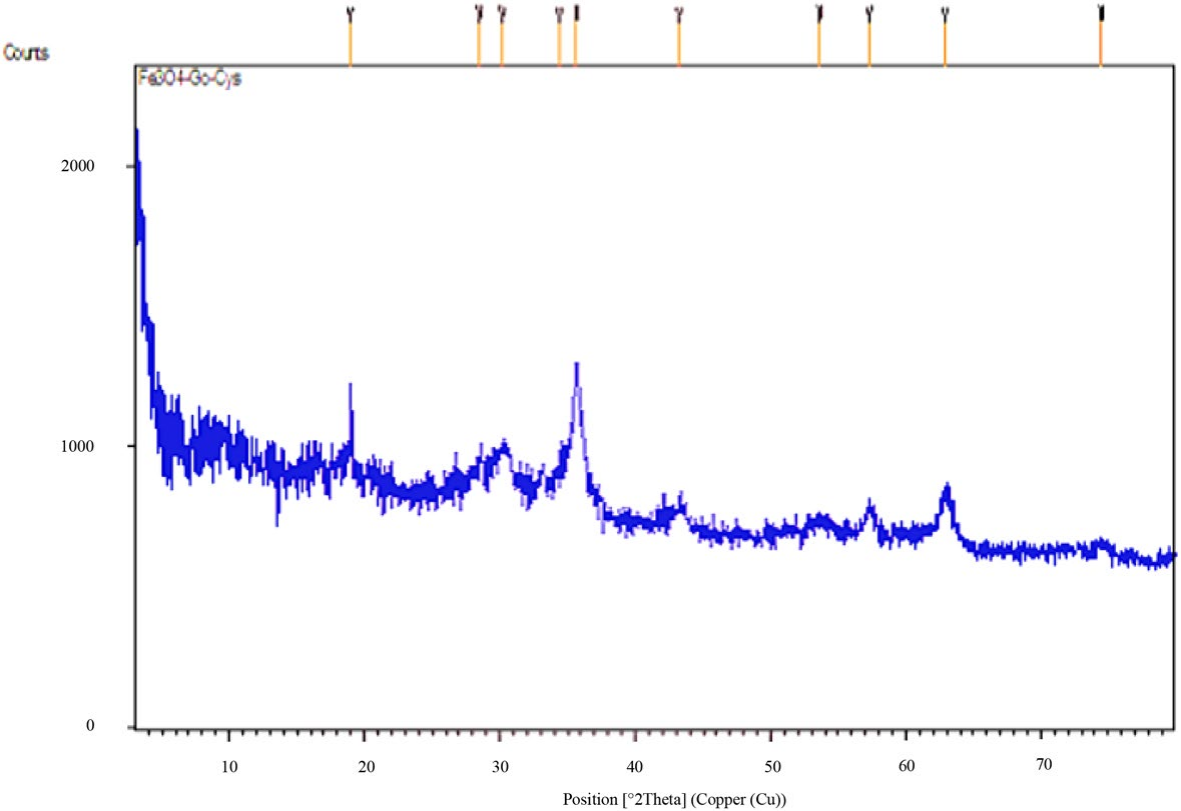
XRD pattern of MNPs·GO-CysA.

XRD analysis of MNPs·GO-CysA clearly indicates the Fe_3_O_4_ spinel structure (Fig. 5). Peaks located at 18.95°, 28.53°, 30.19°, 34.40°, 35.67°, 43.24°, 53.63°, 57.28°, 62.93°, and 74.54° proved the crystallographic structure of Fe_3_O_4_ in the catalyst. Considering the obtained data, the catalyst particles’ size was determined to be 10.4 nm from Scherrer’s equation based on the most intense peak of 2θ = 35.67°.

**Figure 6:**
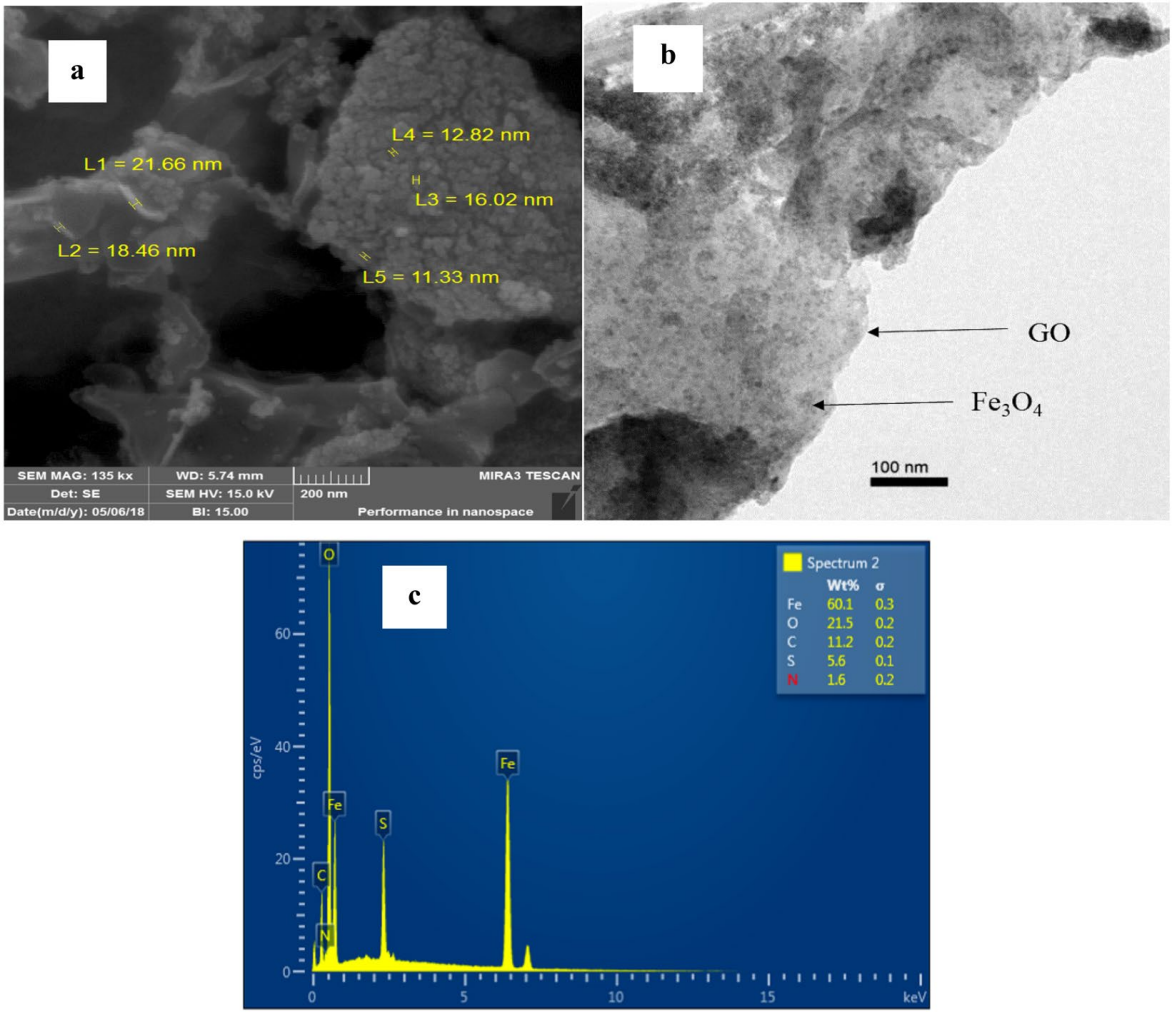
(**a**) SEM, (**b**) TEM, (**c**) EDX analyses of MNPs·GO-CysA.

In order to evaluate the structure, morphology, and size of the catalyst, SEM, TEM and EDX analysis were collected. As can be seen in Fig. 6a**.** the microstructure of MNPs·GO-CysA presented the average size of 16 nm. The TEM micrograph of MNPs·GO-CysA catalyst is shown in Fig. 6b. as shown in Fig. 6b, the sizes of the magnetic Fe_3_O_4_ nanoparticles with tiny particles possessing the spherical morphology were obtained from 10 to 15 nm on a lighter shaded substrate corresponding to the GO sheet. The TEM image of the catalyst (MNPs·GO-CysA) also confirmed that the Fe_3_O_4_ nanoparticles were attached to the surface of graphene oxide free from aggregation. Furthermore, the EDX pattern of the catalyst (Fig. 6c) turned out that MNPs·GO-CysA contains Fe, O, C, N, and S.

CHNS elemental analysis was performed and the results proved the presence of Sulfur atoms in the structure with a scale of 6.68%. The percentage of Sulfur atom in the sample of magnetic GO before the addition of Cysteic acid was determined to be 0.009%. It also appeared that MNPs·GO-CysA contains 1.56% Nitrogen. These results confirm that cysteic acid is successfully attached to the magnetic GO (~ 1.37 mmol g^−1^).

**Figure 7:**
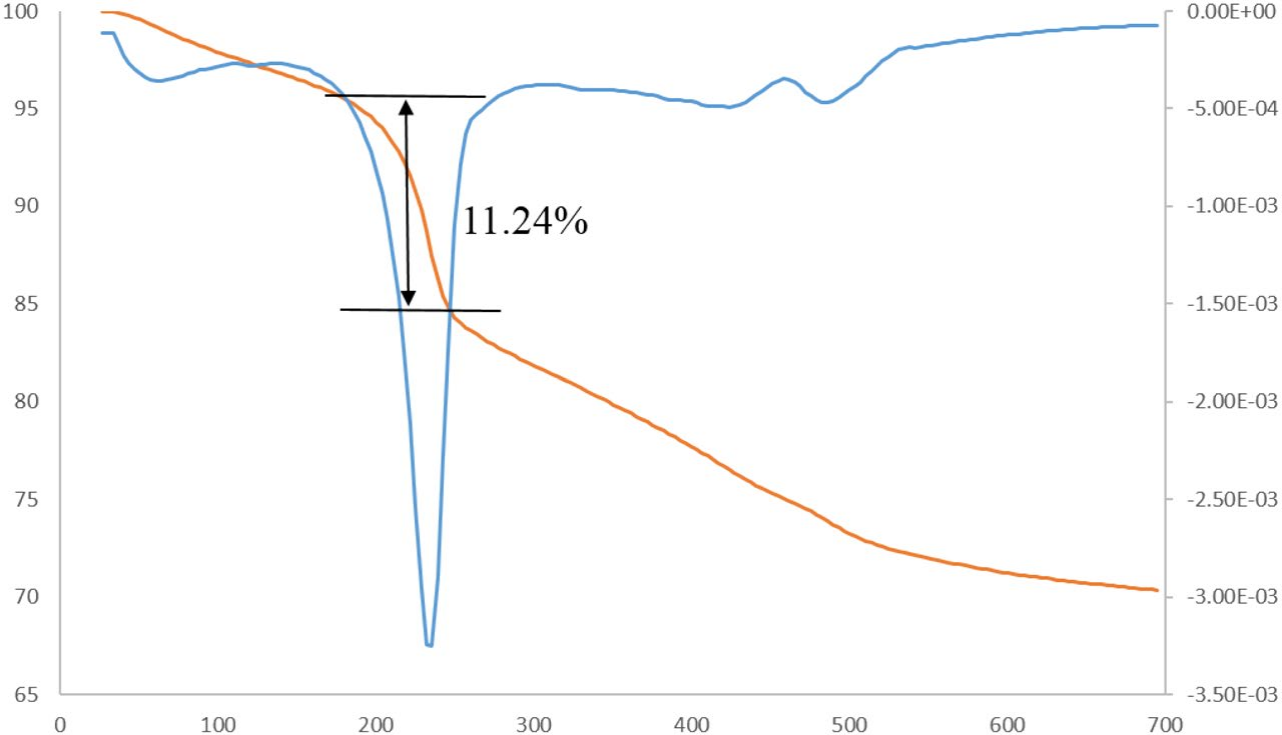
TG-DTG thermograms of MNPs·GO-CysA nanocomposite.

TG-DTG thermograms explain the thermal stability of the MNPs·GO-CysA nanocomposite. All the results derived from TGA analysis are shown in Fig. 7. The first stage of decomposition observed below 180 °C is attributed to physically and chemically trapped water between magnetic GO nanosheets. The second stage of the weight loss, which counted 11.24% between 180 and 220 °C, can be ascribed to the attached organic groups (Cysteic acid) on the surface of magnetic GO nanosheets. Such a relatively high grafting yield suggests successful attachment of Cysteic acid to Fe_3_O_4_/GO surface. The last stage weight loss between 220–600 °C related to the decomposition of graphene oxide nanosheets (the removal of epoxide, hydroxyl, and carboxylic acid surface groups of GO were performed at the beginning of this stage).

**Figure 8:**
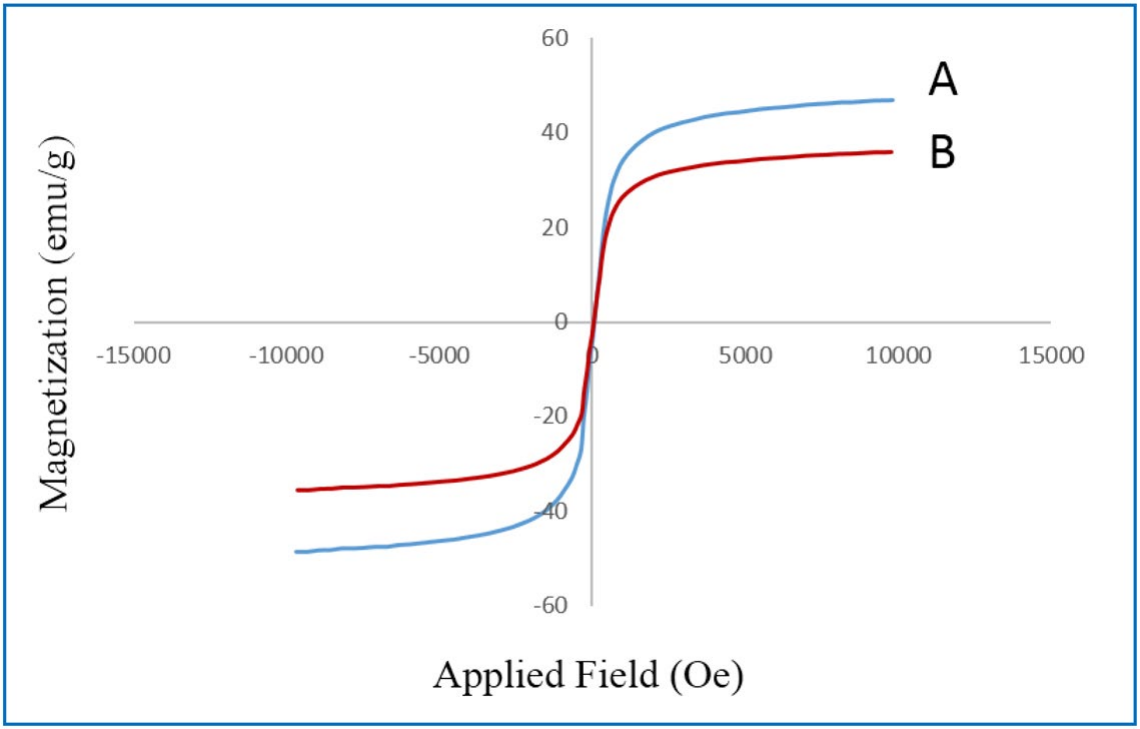
*S*-like curve VSM measurements of Fe_3_O_4_/GO (**A**) and the MNPs·GO-CysA (**B**) nanocomposites.

In the next section, the magnetic behavior of the Magnetic graphene oxide functionalized with cysteic acid nanocomposite has been investigated. In this respect, vibrating sample magnetometer (VSM) measurements were carried out at room temperature for both the Fe_3_O_4_/GO and the MNPs·GO-CysA nanocomposites. The results shown in the Fig. 8**.** indicated that the magnetization value of Fe_3_O_4_/GO (Fig. 8A**)** and MNPs·GO-CysA (Fig. 8B**)** nanocomposites with the *S*-like curve decreases from 46.83 to 35.25 emu g^−1^. This can be attributed to the cysteic acid attached to the Fe_3_O_4_/GO.

Finally, the activity of the magnetically separable acid catalyst was consequently investigated upon characterization in the diver’s derivatives of 4*H*-chromen synthesis. In this regard, to obtain the optimum condition, the three-component reaction between benzaldehyde (**1**), malononitrile (**2**), and 4-hydroxy-6-methyl-2*H*-pyran-2-one (**3**) (molar ratio 1.0:1.1:1.0) was selected as the model reaction and studied in different conditions. The results are summarized in Table 1. In the first step, the reaction was carried out in different solvents including ethanol, water, water–ethanol (3:1), acetonitrile, THF, and solvent-free without any catalysts. The best yield (29%) was obtained in water–ethanol (3:1) in 3 h at room temperature (Table 1, entries 1–6). The Knoevenagel condensation product was formed in quantitative yield as a result of the reaction between benzaldehyde and malononitrile. This result shows that the presence of a catalyst is necessary to improve the desired reaction rate and yield.

**Table 1:** Optimization of the reaction condition for synthesis of 2-amino-3-cyano-4*H*-chromene catalyzed by MNPs·GO-CysA nanocomposite^a^.
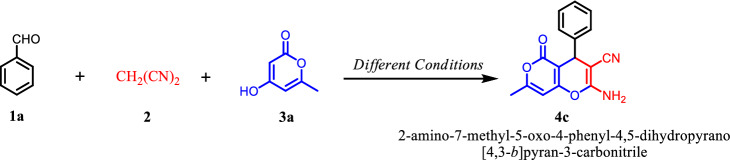

Entry	Cat. (mg)	Temp	Solv	Time	Yield^b^ (%)
1	–	Reflux	EtOH	3 h	14
2	–	Reflux	H_2_O	3 h	16
3	–	Reflux	H_2_O:EtOH (3:1)	3 h	19
4	–	Reflux	CH_3_CN	3 h	Trace
5	–	Reflux	THF	3 h	Trace
6	–	80	Solvent-free	3 h	Trace
7	EDTA **2** mg	Reflux	H_2_O:EtOH (3:1)	3 h	31
8	Cysteic Acid **2** mg	Reflux	H_2_O:EtOH (3:1)	3 h	43
9	GO NPs **2** mg	Reflux	H_2_O:EtOH (3:1)	3 h	27
10	Fe_3_O_4_ NPs **2** mg	reflux	H_2_O:EtOH (3:1)	3 h	23
11	MNPs·Go-CysA **5** mg	Ambient	Solvent-free	3 h	43
12	MNPs·Go-CysA **5** mg	80	Solvent-free	3 h	57
13	MNPs·Go-CysA **5** mg	Ambient	Grinding	3 h	40
14	MNPs·Go-CysA **5** mg	REFLUX	H_2_O:EtOH (3:1)	1.5 h	68
15	MNPs·Go-CysA **7.5** mg	REFLUX	H_2_O:EtOH (3:1)	1.5 h	75
16	MNPs·Go-CysA **10** mg	REFLUX	H_2_O:EtOH (3:1)	1.5 h	79
17	MNPs·Go-CysA **12.5** mg	REFLUX	H_2_O:EtOH (3:1)	1 h	82
18	**MNPs**·**Go-CysA 15 mg**	**REFLUX**	**H**_**2**_**O:EtOH (3:1)**	**30 min**	**92**

^a^Reaction conditions: Benzaldehyd (1a, 1 mmol), malononitrile (2, 1.1 mmol), and 4-hydroxy-6-methyl-2*H*-pyran-2-one (3a, 1 mmol) in the presence of MNPs·GO-CysA nanocomposite and 2 ml of water–ethanol (3:1) as a green solvent. ^b^Isolated Yields.

In the studies of different green catalysts such as ethylenediaminetetraacetic acid on (EDTA), cysteic acid, graphene oxide, and Fe_3_O_4_ nanoparticles on the model reaction, it was found that the best yield of desired product **5e** (43%) was obtained when the cysteic acid was used as the catalyst in ethanol–water under reflux conditions (Table 1, entry 7–10).

Studies showed that acidic reagent plays the main role in the catalytic cycle in these reactions. Therefore, cysteic acid was attached as a green biodegradable amino acid to the magnetic graphene oxide to increase the acidic sites of graphene oxide and to obtain a suitable catalytic activity. Then, the prepared catalyst was used in the model reaction. The reaction was studied under different conditions including running the model reaction at r.t., at 80 °C and using the grinding technique under the solvent-free condition. The desired product was not formed in a suitable yield at any of the mentioned conditions. Interestingly, the product was obtained properly under reflux in water–ethanol (1:3) in the presence of 5 mg of MNPs·Go-CysA nano-catalyst with a yield of 68% after 3 h (Table 1, entries 11–14). Therefore, the effect of catalyst loading on the completion of the reaction was examined in the next experiments (Table 1, entries 15–18). By increasing the catalyst amount from 5 to 15 mg, the reaction yield improved from 68 to 92% at 30 min.

This result clearly shows that the catalyst is effective enough to improve the reaction yield. The active sites of the magnetic nano-catalyst, as a solid acid, can be the acidic functional groups, including the graphene oxide's carboxylic acid group, and the cysteic acid's carboxylic and sulphonic acid groups. These active sites as Bronsted acids improve the reaction yield. The Fe_3_O_4_ nano-particles also can race the reaction up as Lewis acids. However, the optimization results indicate the major active site of the nano-particles to be cysteic acid's functional groups.

Consequently, we developed the optimized reaction condition (15 mg of MNPs·GO-CysA in 2 ml of water–ethanol (3:1) under reflux conditions) for other derivatives of aromatic aldehydes (**1**) and enolizable compound (**3**, **4**, **7**, **10**) for the synthesis of the various derivatives of 2-amino-3-cyano-4*H*-chromenes (**5a–j**, **6a–j**, **8a–j**, **11a–l**). The results have been presented in Tables 2, 3, 4.

**Table 2:** Three-component synthesis of different 2-amino-7-methyl-5-oxo-4-phenyl-4,5-dihydropyrano[4,3-b]pyran-3-carbonitrile (5a-j) and 2-amino-5-oxo-4-phenyl-4,5-dihydropyrano[3,2-c]chromene-3-carbonitrile(6a-j) via condensation of various aldehydes (1), malononitrile (2) and 4-hydroxy-6-methyl-2*H*-pyran-2-one (3)/or 4-hydroxy coumarin (4) in the presence of MNPs·GO-CysA^a^.
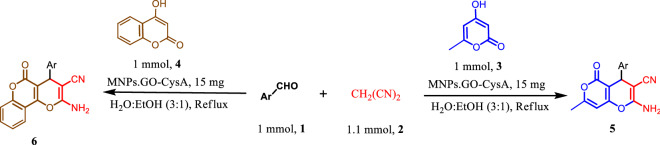

En	Aldehyde	Enolizable compound	Product^b^	Time (min)	Yield^c^(%)	M.P. (°C) Obsd./Lit
1	4-Chlorobenzaldehyde	3	**5a**	15	96	228–230/232^81^
2	2-Chlorobenzaldehyde	3	**5b**	25	93	268–269/266–268^55^
3	4-Nitrobenzaldehyde	3	**5c**	15	94	213–215/210–212^55^
4	3-Nitrobenzaldehyde	3	**5d**	20	95	233–234/234–236^59^
5	Benzaldehyde	3	**5e**	30	92	230–232/235–237^73^
6	Terephthaldehyde	3	**5f**	45	89	253–255/256^29^
7	4-Ethoxybenzaldehyde	3	**5g**	35	93	201–203/224–226^55^
8	4-Methoxybenzaldehyde	3	**5h**	30	91	214–216/209–211^73^
9	3-Methylbenzaldehyde	3	**5i**	50	89	232–234/235–237^73^
10	Thiophen-2-carbaldehyde	3	**5j**	40	91	245–247/240–243^110^
11	4-Chlorobenzaldehyde	4	**6a**	20	98	256–257/258–260^111^
12	2,4-dichlorobenzaldehyde	4	**6b**	35	89	262–264/258–260^112^
13	4-Nitrobenzaldehyde	4	**6c**	14	91	254–256/251–253^73^
14	3-Nitrobenzaldehyde	4	**6d**	25	94	257–259/260–262^113^
15	Benzaldehyde	4	**6e**	25	91	258–260/262–264^111^
16	Terephthaldehyde	4	**6f**	20	86	297–299/305–307^114^
17	4-Methylbenzaldehyde	4	**6g**	25	91	253–255/253–255^73^
18	3-Methylbenzaldehyde	4	**6h**	20	89	253–255/250–252^55^
19	4-Methoxybenzaldehyde	4	**6i**	35	93	233–235/232–234^110^
20	Thiophen-2-carbaldehyde	4	**6j**	35	89	223–225/227–229^110^

^a^Reaction conditions: Aldehyde (1, 1 mmol), Malononitrile (2, 1.1 mmol), 4-hydroxy-6-methyl-2*H*-pyran-2-one (3) /or 4-hydroxy coumarin (4) (1 mmol), and MNPs·GO-CysA (15 mg) at reflux conditions. ^b^All compounds are known and their structures were established from their melting points as compared with authentic samples or literature values. ^c^Isolated yield.

**Table 3:** Three-component synthesis of different 2-amino-7,7-dimethyl-5-oxo-4-aryl-5,6,7,8-tetrahydro-4*H*-chromene-3-carbonitrile (**8a**–**j**) via condensation of various aldehydes (1), malononitrile (2) and dimedone (7) in the presence of MNPs·GO-CysA^a^.
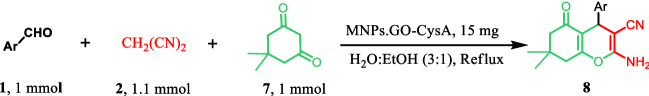

En	Aldehyde	Enolizable compound	Product^b^	Time (min)	Yield^c^ (%)	M.P (°C) Obsd./Lit
1	2-Chlorobenzaldehyde	7	**8a**	15	95	217–219/213–215^73^
2	3-Nitrobenzaldehyde	7	**8b**	15	90	210–211/210–212^55^
3	2- Nitrobenzaldehyde	7	**8c**	20	95	218–221/220–222^55^
4	Benzaldehyde	7	**8d**	10	90	235–237/231–232^110^
5	4-Methoxybenzaldehyde	7	**8e**	15	93	212–214/210–212^112^
6	4-Methoxybenzaldehyde	7	**8f**	15	91	195–197/200–202^73^
7	2,4-dichlorobenzaldehyde	7	**8g**	15	90	221–223/218–220^115^
8	Terphthaldehyde	7	**8h**	30	89	208–211
9	Vanillin	7	**8i**	20	89	240–242/239–241^73^
10	Thiophen-2-carbaldehyde	7	**8j**	30	89	219–220/222–224^116^

^a^Reaction conditions: Aldehyde (1, 1 mmol), Malononitrile (2, 1.1 mmol), dimedone (7, 1 mmol), and MNPs·GO-CysA (15 mg) at reflux conditions. ^b^All compounds are known and their structures were established from their melting points as compared with authentic samples or literature values. ^c^Isolated yield.

**Table 4:** Four-component synthesis of different 6′-amino-3′-methyl-2-oxo-1′*H*-spiro[indoline-3,4′-pyrano[2,3-c]pyrazole]-5′-carbonitrile (11a-l) via condensation of various isatin (9), malononitrile (2) and 3-methyl-1*H*-pyrazol-5(4*H*)-one (10) in the presence of MNPs·GO-CysA^a^.
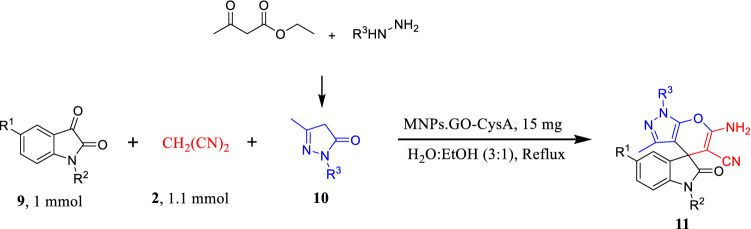

En	Isatin	R	Product^b^	Time (min)	Yield^c^ (%)	M.P (°C) Obsd./Lit
1	Isatin	H	**11a**	20	95	269–271/286–287^67^
2	*N*-Allyl Isatin	H	**11b**	25	91	240–242/244–246^30^
3	*N*-(4-Nitrobenzyl) Isatin	H	**11c**	30	88	272–273/271–273^45^
4	*N*-Methyl Isatin	H	**11d**	30	89	261–263/262–264^30^
5	*N*-Ethyl Isatin	H	**11e**	20	90	283–285/285–287^30^
6	*N*-Propargyl Isatin	H	**11f**	25	90	258–259/258–262^30^
7	*N*-Benzyl Isatin	H	**11g**	25	91	232–233/228–232^30^
8	Isatin	Ph	**11h**	25	94	223–225/228–230^41^
9	*N*-Allyl Isatin	Ph	**11i**	25	90	213–215/218–220^117^
10	*N*-Methyl Isatin	Ph	**11j**	15	98	226–228/220–222^118^
11	*N*-Ethyl Isatin	Ph	**11k**	20	91	213–215/210–212^117^
12	*N*-Benzyl Isatin	Ph	**11l**	30	89	231–233/228–230^119^

^a^Reaction conditions: Isatin (9, 1 mmol), Malononitrile (2, 1.1 mmol), hydrazine compounds (1 mmol), ethyl acetoacetate (1 mmol), and MNPs·GO-CysA (15 mg) at reflux conditions. ^b^All compounds are known and their structures were established from their melting points as compared with authentic samples or literature values. ^c^Isolated yield.

In the next step, to demonstrate the scope of the present protocol, the optimized reaction conditions were examined for Dimedone **7.** Dimedone as a cyclic 1,3-dicetone compounds (p*K*a = 5.23) required shorter reaction time compared to the 4-hydroxy-pyrane and 4-hydroxy-cumarin for the synthesis of 2-amino-7,7-dimethyl-5-oxo-4-aryl-5,6,7,8-tetrahydro-4*H*-chromene-3-carbonitrile (**8a–j**) in good to excellent yields.

It is noteworthy that in all processes of 2-amino-3-cyano-4*H*-chromene derivatives (5, 6, 8) syntheses, the reaction of aromatic aldehydes which possessed electron-withdrawing groups are shown to be faster than the reaction of aromatic aldehydes with electron-donating groups. Additionally, the reaction was proceeding with heterocyclic aldehydes, and the desired products were obtained in good yields.

Finally, to demonstrate the effectiveness and efficiency of this new method, the optimized conditions were developed for four-component one-pot condensation of ethyl acetate, hydrazine hydrate/or phenyl hydrazine, malononitrile, and isatin. It should be pointed out that the reaction proceeded at reflux conditions to give the purely expected 6′-amino-3′-methyl-2-oxo-1′*H*-spiro[indoline-3,4′-pyrano[2,3-c]pyrazole]-5′-carbonitrile (**11a–l**) in quantitative yields in very short reaction time (Table 4).

In all studies, the reaction catalyst was simply separated from the main product employing an external magnet after cooling the mixture to r.t. and the precipitated product was filtered out of the reaction mixture.

**Figure 9:**
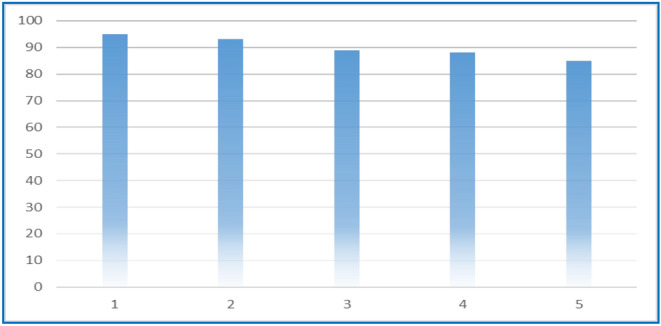
The recycling capability of MNPs·GO-CysA nano catalyst in the synthesis of spiro 4*H*-chromene (**11a**) under optimized conditions.

**Figure 10:**
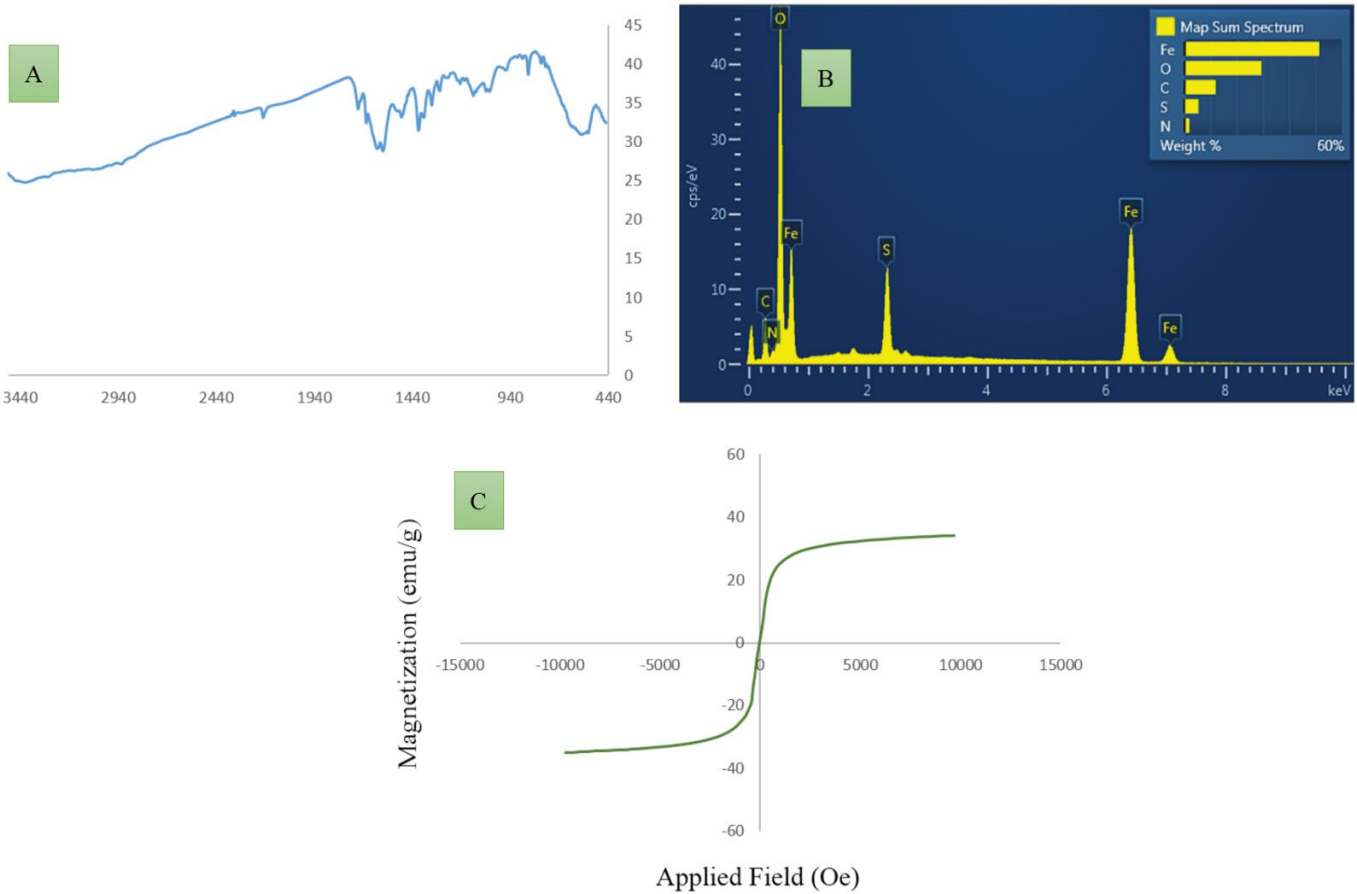
The FTIR spectrums (**A**) and EDX analysis (**B**) and (**C**) VSM curve of recoverd MNPs·GO-CysA after five runs.

Due to the importance of using heterogeneous catalysts in industrial processes, we studied the recyclability of the MNPs·GO-CysA nano-catalyst by using it in repeatedly five runs reactions for product **11a** under the optimized reaction conditions. After each reaction cycle, the superparamagnetic catalyst was separated by an external magnetic field and washed twice with hot deionized water (5 mL), once with 5 mL ethanol, dried in an oven at 70 °C, and reused in the model reaction. Summarized results in Fig. 9. implied that a significant reduction in catalytic efficiency was not observed after 5 runs. The strong covalence interaction of cysteic with the GO surface could be the reason for the repetitive use of the catalyst in a greater number of catalytic runs with high efficiency. Eventually, the comparison of the results of FT-IR, EDX, and VSM analysis of the recycled catalyst after five cycles revealed that there are no significant structural changes occurred at the nanoparticle surface (Fig. 10).

In order to evaluate the catalytic efficiency of MNPs·Go-CysA nano-catalyst, we compared the advantages of this catalyst with some other previously reported catalysts for the synthesis of 4*H*-chromenes. The comparison proved that the catalyst possesses higher activity compared to other catalysts (Table 5).

**Table 5:** Comparative synthesis of compound **5e**, **6h**, **8d**, and **11h** using the recently reported methods versus the present method.

En	Catalyst	Catalyst loading, Time, Yield, Tem., Solvent	References
1	AcONH_4_	10 mol%, 10 min, 94%, r.t, EtOH	^120^^a^
2	Piperidine	2 drops, 6 h, 92%, Reflux, EtOH	^59a^
3	MNPs·GO-CysA	15 mg, 30 min, 94%, Reflux, H_2_O:EtOH	This work^a^
4	(2-Aminomethyl)Phenol/ Hydroxyapatite	1.5 mol%, 30 min, 78%, Reflux, H_2_O	^121b^
5	POPINO	5 mol%, 10 min, 97%, Reflux, H_2_O	^51b^
6	Visible Light, 20 W	–, 1.6 h, 87%, –, S-F	^122b^
7	PEI@Si–MNP	5 mg, 55 min, 89%, Reflux, Ethylene Glycol/water	^69b^
8	MNPs·GO-CysA	15 mg, 25 min, 91%, Reflux, H_2_O:EtOH	This work^b^
9	IL-HSO_4_@SBA-15	2 mol%, 2 h, 94%, 45 °C, H_2_O	^42c^
10	Zn_4_O(H_2_N-TA)_3_	40 mg, 5 h, 95%, 60 °C, S-F	^31c^
11	Piperidinium Acetate	10 mol%, 30 min, 92%, r.t, H_2_O	^60c^
12	MNPs·GO-CysA	15 mg, 10 min, 90%, Reflux, H_2_O:EtOH	This work^c^
13	PBBS	20 mg, 4 h, 80%, r.t, CH_3_CN	^75d^
14	Bmim(OH)/ 20 mol% Chitosan	5 ml, 3 h, 92%, r.t, -	^82d^
15	ZnS nanoparticles, Sonicate	10 mol%, 13 min, 96%, r.t, H_2_O	^32d^
16	4-DMAP	10 mol%, 60 min, 83%, 60 °C, EtOH	^58d^
17	Bovine Serum Albumin	60 mg, 30 min, 98%, r.t, H_2_O:EtOH	^84d^
18	MNPs·GO-CysA	15 mg, 20 min, 95%, Reflux, H_2_O:EtOH	This work^d^

^a^Obtained results for the synthesis of compound 5e*.* ^b^Obtained results for the synthesis of compound 6 h*.* ^c^Obtained results for the synthesis of compound 8d. ^d^Obtained results for the synthesis of compound 11 h.

## Conclusions

In summary, we have successfully developed a solid acid supported magnetic graphene oxide catalyzed reaction of an enolizable compound, active methylene nitriles, and aldehydes, which provides rapid and efficient access to different 4*H*-chromene derivatives. Cysteic acid has been incorporated on the magnetic GO successfully and properly dispersed between GO sheets. We have demonstrated that MNPs·GO-CysA acts as a non-hazardous, efficient, reusable, and convenient catalytic system for the synthesis of a wide range of 4*H*-chromenes in water/ethanol as a green solvent. Additionally, the presented catalyst was easily removed from the reaction mixture by means of an external magnet and reused several times with little loss of activity. It can be seen that simplicity in preparation of the present catalyst, efficient recyclability and reusability of the catalyst, short reaction times, and high yields of the products can be considered as outstanding characteristics of the present protocol.

## Experimental

### Materials

All the materials which were used in our experiments including reagents and solvents were purchased from Merck or Sigma Aldrich. The materials were used without further purification, while benzaldehyde was used freshly after distillation. We used our University’s Bruker (Avance DRX-500) spectrometer in order to have our products been checked via ^1^H NMR and ^13^C NMR spectrometry using CDCl_3_ as solvent at room temperature. Chemical shifts from the initial standard tetramethylsilane are reported in parts per million (ppm). Using an ABB Bomem MB100 FTIR spectrophotometer, the samples were tested, the results of which are reviewed. CHNS analysis was done by LECO Truspec. Scanning electron microscopy (SEM) was performed on VEGA\\TESCAN-LMU. An energy dispersive detector (EDS) coupled to the microscope was used to identify chemical elements of the prepared catalyst. X-ray diffraction (XRD) pattern was recorded on APD 2000 using Cu Kα radiation (50 kV, 150 mA) in the range 2θ = 10–120°. CHN analysis was done by LECOTruspec.

### Preparation of catalyst

Graphene oxide (GO) was prepared via a modified Hummers method^123^. The general procedure for preparation of magnetic GO: 50 mL aqueous solution of 4 mmol FeCl_3_·6H_2_O and 2 mmol FeCl_2_·4H_2_O was prepared. The pH of the solution was adjusted to pH = 4 using a NaOH solution (1 M). A Graphene oxide solution was prepared by dispersing 27.5 mg GO in 20 mL water. The Graphene oxide solution was gradually added to the first solution and stirred for 30 min. After that, a sufficient amount of NaOH (1 M) was added to the solution until the pH was adjusted to 10. The reaction was then stirred for 1 h and the resulting precipitate was separated by means of a magnet and washed with DI water and ethanol three times. The resulting precipitate was dried in an oven at 70 °C^124^.

In order to attach L-cysteic acid to the prepared magnetic GO, a mixture of 200 mg of magnetic GO, 50 mg of l-cysteic acid, and 5 ml ethanol was placed in a round bottom flask under stirring for 24 h at room temperature. The obtained precipitate was washed with water and ethanol and dried at 70 °C.

### Synthesis of 4*H*-chromene derivatives

A glass vial was successively charged with different enolizable compounds (1 mmol), aldehydes (1 mmol), and active methylene nitrile (1.1 mmol) in the presence of MNPs·GO-CysA (15 mg), in water–ethanol (3:1, 2 mL) at reflux temperature. The reaction mixture was stirred for the appropriate time brought in Tables 2, 3, and 4. After reaction completion, which was controlled by Thin Layer Chromatography (TLC) test (using EtOAc/ n-Hexane, 1:3 as solvent), the catalyst was separated by a magnet, and the obtained solid product was filtered. In the case of impurities, the obtained product was recrystallized from ethanol.

### Synthesis of Spiro 4*H*-chromene derivatives

A glass vial was successively charged with hydrazine monohydrate (1.1 mmol), ethyl acetoacetate (1 mmol), MNPs·GO-CysA (15 mg), water–ethanol (3:1, 2 mL) and stirred at reflux conditions for 5 min. Then isatin derivatives (1 mmol), methylene reagent (malononitrile, 1.1 mmol) were added to reaction mixture and stirred for the appropriate time brought in Table 4. After reaction completion, which was controlled by Thin Layer Chromatography (TLC) test (using EtOAc/ n-Hexane, 1:3 as solvent) and the reaction color change from red to white, the catalyst was separated by a magnet, and the obtained solid product was filtered. In the case of impurities, the obtained product was recrystallized from ethanol.

## Supplementary information

Supplementary Information.
