# Supplementary material for: Cysteic acid grafted to magnetic graphene oxide as a promising recoverable solid acid catalyst for the synthesis of diverse 4H-chromene
Source: Sci Rep. 2020 Dec 1;10:20968. doi: 10.1038/s41598-020-77872-8 (PMC7708834; doi:10.1038/s41598-020-77872-8)
Supplement: Supplementary file 1 — Supplementary Information. [file 41598_2020_77872_MOESM1_ESM.docx]

***Supporting Information***

**Cysteic acid grafted to magnetic graphene oxide as a promising recoverable solid acid catalyst for the synthesis of diverse 4*H*-chromene**

Firouz Matloubi Moghaddam*, Mohammad Eslami, and Golfamsadat Hoda

Laboratory of Organic Synthesis and Natural Products, Department of Chemistry, Sharif University of Technology, Tehran, Iran.

Correspondence Firouz Matloubi Moghaddam, Laboratory of Organic Synthesis and Natural Products, Department of Chemistry, Sharif University of Technology, Azadi Street, PO Box 111559516, Tehran, Iran Email: matloubi@sharif.edu. Funding information Sharif University of Technology (SUT).

| ***CONTENTS***  ^1^HNMR, ^13^CNMR, and FT-IR Characterization data for products 5g, 6b, 8c, 11a, 11d, 11f, 11g, 11j, 11k, 11l……………………………………………………………………………..……..…… II-XXI |
| --- |

**Characterization data of products**

**Data for 2-amino-4-(4-ethoxyphenyl)-7-methyl-5-oxo-4*H*,5*H*-pyrano[4,3-*b*]pyran-3-carbonitrile 5g:**

m.p 201-203 °C; ^1^H NMR (500 MHz, CDCl_3_) δ 1.40 (3H, t, *J* = 7.0 Hz, CH_3_), 2.24 (3H, s, CH_3_), 4.20 (2H, q, CH_2_), 4.43 (1H, s, CH), 4.71 (2H,br s, NH_2_), 5.90 (1H, s, CH), 6.85 (2H, d, *J* = 8.0 Hz, H-Ar), 7.20 (2H, d, *J* = 8.5 Hz, H-Ar); ^13^C NMR (125 MHz, DMSO-d_6_) δ 14.62, 19.27, 35.57, 58.14, 62.90, 97.84, 101.05, 114.17, 119.43, 128.57, 135.36, 157.53, 157.90, 158.02, 160.35,165.74; IR (KBr) *ν* (cm^-1^) 3404, 3331, 3216, 2988, 2973, 2889, 2199, 1699, 1673, 1646, 1610, 1511,1378, 1045, 784.

**Data for 2-amino-7-methyl-4-(3-nitrophenyl)-5-oxo-4*H*,5*H*-pyrano[4,3-*b*]pyran-3-carbonitrile 6b:**

m.p 262-264 °C; ^1^H NMR (500 MHz, DMSO-d_6_) δ 4.99 (1H, s,CH), 7.37 (2H, m, H-Ar), 7.50 (4H, m, H-Ar), 7.59 (1H, s, , H-Ar), 7.74 (1H, t, *J* = 7.5 Hz, H-Ar), 7.91 (1H, d, *J* = 7.5 Hz, H-Ar); ^13^C NMR (125 MHz, DMSO-d_6_) δ 33.96, 55.14, 102.50, 112.84, 116.57, 118.70, 122.46, 124.65, 127.87, 128.76, 132.41, 133.04, 133.23, 133.40, 139.44, 152.36, 154.18, 158.15, 159.41 ; IR (KBr) *ν* (cm^-1^) 3462, 3302, 3174, 3062, 2197, 1698, 1655, 1390, 754.

**Data for 2-amino-7,7-dimethyl-4-(2-nitrophenyl)-5-oxo-5,6,7,8-tetrahydro-4H-chromene-3-carbonitrile 8c:**

m.p 198-200 °C; ^1^H NMR (500 MHz, DMSO-d_6_) δ 0.88 (3H, s, CH_3_), 1.01 (3H, s, CH_3_), 2.10 (2H, ABq, CH_2_), 2.50 (2H, ABq, CH_2_), 4.93 (1H, s, CH), 7.22 (2H, br s, NH_2_), 7.36 (1H, d, *J* = 7.5 Hz, H-Ar), 7.43 (1H, t, *J* = 8.0 Hz, H-Ar), 7.67 (1H, t, *J* = 7.5 Hz, H-Ar), 7.82 (1H, d, *J* = 8.0 Hz, H-Ar); ^13^C NMR (125 MHz, DMSO-d_6_) δ 26.89, 28.45, 31.80, 49.96, 56.83, 99.82, 111.81, 119.30, 127.47, 128.23, 129.47, 129.99, 132.13, 141.60, 158.70, 163.18, 197.58; IR (KBr) *ν* (cm^-1^) 34740 3329, 3248, 2198, 1688, 1659, 1597,1528, 1359, 1218, 639.

**Data for 6'-amino-3'-methyl-2-oxo-1'*H*-spiro[indoline-3,4'-pyrano[2,3-*c*]pyrazole]-5'-carbonitrile 11a:**

m.p 269-271 °C; ^1^H NMR (500 MHz, DMSO-d_6_) δ 1.53 (3H, s, CH_3_), 6.59 (1H, d, *J* = 7.5 Hz, H-Ar), 7.00 (1H, t, *J* = 7.5 Hz, H-Ar), 7.04 (1H, d, *J* = 7.5 Hz, H-Ar), 7.25 (3H, m, H-Ar), 10.60 (1H, br s, NH), 12.29 (1H, br s, NH); ^13^C NMR (125 MHz, DMSO-d_6_) δ 9.06, 47.20, 55.19, 95.41, 109,70, 118.78, 122.54, 124.54, 128.93, 132.71, 134.77, 141.53, 155.28, 162.50, 178.06; IR (KBr) *ν* (cm^-1^) 3380, 3330, 3131, 2930, 2890, 2190, 1704, 1642, 1606, 1406, 1374, 759.

**Data for 6'-amino-1,3'-dimethyl-2-oxo-1'*H*-spiro[indoline-3,4'-pyrano[2,3-*c*]pyrazole]-5'-carbonitrile 11d:**

m.p 261-263 °C; ^1^H NMR (500 MHz, DMSO-d_6_) δ 1.45 (3H, s, CH_3_), 3.12 (3H, s, CH_3_), 7.10 (3H, m, H-Ar), 7.28 (2H, br s, NH_2_), 7.36 (1H, t, *J* = 5.5 Hz, H-Ar), 12.29 (1H, br s, NH); ^13^C NMR (125 MHz, DMSO-d_6_) δ 9.09, 26.37, 47.01, 54.82, 96.28, 108.71, 118.67, 123.25, 124.19, 129.09, 131.94, 134.80, 142.98, 155.27, 162.66, 176.35; IR (KBr) *ν* (cm^-1^) 3394, 3343, 3151, 2965, 2808, 2184, 1712, 1644, 1585, 1413, 1378, 756.

**Data for 6'-amino-1,3'-dimethyl-2-oxo-1'*H*-spiro[indoline-3,4'-pyrano[2,3-*c*]pyrazole]-5'-carbonitrile 11f:**

m.p 256-259 °C; ^1^H NMR (500 MHz, DMSO-d_6_) δ 1.48 (3H, s, CH_3_), 3.26 (1H, s, CH), 4.60 (2H, ABq, CH_2_), 7.14 (2H, m, H-Ar), 7.20 (1H, d, *J* = 8.0 Hz, H-Ar), 7.33 (2H, br s, NH_2_), 7.39 (1H, m, H-Ar), 12.37 (1H, br s, NH); ^13^C NMR (125 MHz, DMSO-d_6_) δ 9.13, 11.12, 20.49, 41.23, 46.92, 55.13, 95.27, 108.90, 118.58, 123.02, 124.40, 129.05, 132.02, 134.70, 142.47, 155.33, 162.55, 176.36; IR (KBr) *ν* (cm^-1^) 3388, 3333, 3176, 2974, 2963, 2929, 2195, 1700, 1644, 1604, 1406, 761, 689.

**Data for 6'-amino-1-benzyl-3'-methyl-2-oxo-1'H-spiro[indoline-3,4'-pyrano[2,3-c]pyrazole]-5'-carbonitrile 11g:**

m.p 232-234 °C; ^1^H NMR (500 MHz, DMSO-d_6_) δ 1.35 (3H, s, CH3), 4.96 (2H, ABq, CH_2_), 7.03 (1H, d, *J* = 8.5 Hz, H-Ar), 7.07 (1H, d, *J* = 7.0 Hz, H-Ar), 7.12 (1H, d, *J* = 7.0 Hz, H-Ar), 7.27 (2H, t, *J* = 7.0 Hz, H-Ar), 7.33 (4H, m, H-Ar), 7.41 (1H, d, *J* = 7.5 Hz, H-Ar), 12.34 (1H, br s, NH); ^13^C NMR (125 MHz, DMSO-d_6_) δ 9.05, 43.22, 46.82, 54.81, 95.22, 99.54, 109.36, 118.76, 123.34, 124.49, 127.57, 128.59, 128.99, 131.90, 134.81, 136.17, 142.06, 155.32, 162.68, 176.71; IR (KBr) *ν* (cm^-1^) 3399, 3294, 3243, 3157, 2975, 2943, 2868, 2202, 1710, 1640, 1595, 1406, 758.

**Data for 6'-amino-1,3'-dimethyl-2-oxo-1'-phenyl-1'H-spiro[indoline-3,4'-pyrano[2,3-c]pyrazole]-5'-carbonitrile 11j:**

m.p 226-228 °C; ^1^H NMR (500 MHz, DMSO-d_6_) δ 1.40 (3H, s, CH_3_), 3.25 (3H, s, CH_3_), 7.12 (1H, t, *J* = 7.0 Hz, H-Ar), 7.16 (1H, d, *J* = 8.0 Hz, H-Ar), 7.36 (1H, t, *J* = 7.5 Hz, H-Ar), 7.40 (1H, t, *J* = 7.5 Hz, H-Ar), 7.53 (2H, t, *J* = 8.0 Hz, H-Ar), 7.65 (2H, br s, NH_2_), 7.80 (2H, d, *J* = 8.0 Hz, H-Ar); ^13^C NMR (125 MHz, DMSO-d_6_) δ 11.67, 25.23, 47.88, 56.16, 96.28, 109.87, 117.95, 120.25, 122.68, 125.89, 126.54, 129.36, 132.15, 137.26, 141.58, 144.01, 144.95, 160.98, 177.26; IR (KBr) *ν* (cm^-1^) 3461, 3296, 3177, 3070, 2951, 2921, 2196, 1700, 1656, 1614, 1468, 1391, 1331, 755.

**Data for 6'-amino-1-ethyl-3'-methyl-2-oxo-1'-phenyl-1'H-spiro[indoline-3,4'-pyrano[2,3-c]pyrazole]-5'-carbonitrile 11k:**

m.p 213-215 °C; ^1^H NMR (500 MHz, DMSO-d_6_) δ 1.20 (3H, t, *J*=7.5 Hz, CH_3_), 1.48 (3H, s, CH_3_), 3.80 (2H, m, CH_2_), 7.11 (1H, t, *J* = 7.5 Hz, H-Ar), 7.20 (1H, d, *J* = 8.0 Hz, H-Ar), 7.24 (1H, d, *J* = 7.5 Hz, H-Ar), 7.38 (2H, m, H-Ar), 7.53 (2H, t, *J* = 8.0 Hz, H-Ar), 7.63 (2H, br s, NH_2_), 7.79 (2H, d, *J* = 8.0 Hz, H-Ar); ^13^C NMR (125 MHz, DMSO-d_6_) δ 11.71, 12.56, 34.67, 47.38, 56.08, 96.16, 108.90, 117.77, 120.06, 120.28, 123.20, 124.82, 126.60, 129.43, 131.65, 137.27, 141.99, 143.94, 145.05, 161.11, 175.43; IR (KBr) *ν* (cm^-1^) 3368, 3307, 3195, 2991, 2931, 2882, 2197, 1711, 1651, 1604, 1379, 749.

**Data for 6'-amino-1-benzyl-3'-methyl-2-oxo-1'-phenyl-1'*H*-spiro[indoline-3,4'-pyrano[2,3-*c*]pyrazole]-5'-carbonitrile 11l:**

m.p 231-233 °C; ^1^H NMR (500 MHz, DMSO-d_6_) δ 1.36 (3H, s, CH_3_), 5.02 (2H, ABq, CH_2_), 7.10 (2H, m, H-Ar), 7.32 (6H, m) , H-Ar, 7.45 (2H, d, *J* = 7.0 Hz), 7.53 (2H, t, *J* = 7.5 Hz, H-Ar), 7.69 (2H, br s, NH), 7.45 (2H, d, *J* = 8.0 Hz, H-Ar); ^13^C NMR (125 MHz, DMSO-d_6_) δ 11.65, 43.38, 47.59, 55.97, 96.18, 109.54, 118.05, 120.11, 120.32, 123.47,124.86, 126.64, 127.64, 128.62, 129.38, 129.58, 131.42, 136.04, 137.27, 142.18, 143.93, 145.07, 161.28, 176.20; IR (KBr) *ν* (cm^-1^) 3358, 3320, 3195, 2906, 2855, 2201, 1705, 1663, 1523, 1397, 748.
